# The Effects of Suicide Exposure on Mental Health Outcomes Among Post-9/11 Veterans: Protocol for an Explanatory, Sequential, Mixed Methods Study

**DOI:** 10.2196/51324

**Published:** 2023-09-26

**Authors:** Nina A Sayer, David B Nelson, Jaimie L Gradus, Rebecca K Sripada, Maureen Murdoch, Alan R Teo, Robert J Orazem, Julie Cerel

**Affiliations:** 1 Center for Care Delivery and Outcomes Research, Minneapolis VA Healthcare System Minneapolis, MN United States; 2 Department of Psychiatry and Behavioral Sciences, University of Minnesota Minneapolis, MN United States; 3 Department of Medicine, University of Minnesota Minneapolis, MN United States; 4 Department of Epidemiology, School of Public Health, Boston University Boston, MA United States; 5 Center for Clinical Management Research, VA Ann Arbor Healthcare System Ann Arbor, MI United States; 6 Department of Psychiatry, University of Michigan Ann Arbor, MI United States; 7 Section of General Internal Medicine, Minneapolis VA Health Care System Minneapolis, MN United States; 8 Center to Improve Veteran Involvement in Care, VA Portland Health Care System Portland, OR United States; 9 Department of Psychiatry, Oregon Health & Science University Portland, OR United States; 10 College of Social Work, University of Kentucky Lexington, KY United States

**Keywords:** veterans, suicide, death, posttraumatic stress disorder, bereavement, health services

## Abstract

**Background:**

The toll associated with suicide goes well beyond the individual who died. This study focuses on a risk factor for veteran suicide that has received little previous empirical attention—exposure to the suicide death of another person.

**Objective:**

The study’s primary objective is to describe the mental health outcomes associated with suicide exposure among veterans who served on active duty after September 2001 (“post-9/11”). The secondary objective is to elucidate why some veterans develop persistent problems following suicide exposure, whereas others do not.

**Methods:**

This is an explanatory, sequential, mixed methods study of a nationally representative sample of post-9/11 veterans enrolled in Department of Veterans Affairs (VA) health care. Our sampling strategy was designed for adequate representation of female and American Indian and Alaska Native veterans to allow for examination of associations between suicide exposure and outcomes within these groups. Primary outcomes comprise mental health problems associated with trauma and loss (posttraumatic stress disorder and prolonged grief disorder) and suicide precursors (suicidal ideation, attempts, and planning). Data collection will be implemented in 3 waves. During wave 1, we will field a brief survey to a national probability sample to assess exposure history (suicide, other sudden death, or neither) and exposure characteristics (eg, closeness with the decedent) among 11,400 respondents. In wave 2, we will include 39.47% (4500/11,400) of the wave-1 respondents, stratified by exposure history (suicide, other sudden death, or neither), to assess health outcomes and other variables of interest. During wave 3, we will conduct interviews with a purposive subsample of 32 respondents exposed to suicide who differ in mental health outcomes. We will supplement the survey and interview data with VA administrative data identifying diagnoses, reported suicide attempts, and health care use.

**Results:**

The study began on July 1, 2022, and will end on June 30, 2026. This is the only national, population-based study of suicide exposure in veterans and the first one designed to study differences based on sex and race. Comparing those exposed to suicide with those exposed to sudden death for reasons other than suicide (eg, combat) and those unexposed to any sudden death may allow for the identification of the common and unique contribution of suicide exposure to outcomes and help seeking.

**Conclusions:**

Integrating survey, qualitative, and VA administrative data to address significant knowledge gaps regarding the effects of suicide exposure in a national sample will lay the foundation for interventions to address the needs of individuals affected by a suicide death, including female and American Indian and Alaska Native veterans.

**International Registered Report Identifier (IRRID):**

DERR1-10.2196/51324

## Introduction

### Background

Suicide is a public health problem that disproportionately affects US veterans. Veterans are 1.5 times more likely to die by suicide than members of the general population, after adjusting for age and sex [1]. Adjusting for age, female veterans are 2.1 times more likely to die by suicide than female nonveterans [1]. Suicide rates are particularly high in the year following military separation and may remain elevated for at least 6 years following separation [2,3].

Groups at increased risk of suicide may also be at increased risk of *suicide exposure*, defined as knowing someone who has died by suicide. It has been estimated that for each person who dies by suicide, 135 others may be exposed, and up to 50% of them may be intimately and directly affected [4,5]. Those exposed to a suicide death are at increased risk for psychiatric disorders, disordered grief, physical disorders, impaired social functioning, and their own fatal and nonfatal suicide behavior compared with those who are unexposed [6-9]. Consequently, suicide prevention strategies recommend *postvention,* which is the provision of services and support to those affected by a suicide death to facilitate their healing [10]. This study characterizes the postvention needs of veterans during the first 6 years after military separation.

Suicide exposure among military populations is an understudied risk factor for suicide. A scoping review of studies based on military samples identified 6 empirical studies of exposure to suicide death and one study of exposure to suicide attempts [11]. Suicide exposure ranged from 47.1% in a sample of 931 veterans living in Kentucky [12] to 65.4% in a sample of 971 active-duty National Guard personnel in Utah and Idaho [13]. Consistent with studies based on civilian samples, studies of suicide exposure in military samples reported high frequencies of mental health disorders, including posttraumatic stress disorder (PTSD) and disordered grief; suicidal ideation; and suicide attempts among those exposed to suicide compared with those who were unexposed [11].

These studies established that suicide exposure is all too common among military populations. However, they leave unanswered questions foundational to preventing and treating mental health and psychosocial problems among those affected by a suicide death. The studies of suicide exposure in military populations were based on geographically distinct or small samples that did not include a sufficient number of female and racial and ethnic minority individuals to examine potentially critical differences based on sex and race. American Indian and Alaska Native individuals serve in the US military at the highest rate per capita compared with other US racial groups and have the highest suicide death rate, yet American Indian and Alaska Native veterans are not represented in these studies [14,15]. Another limitation is regarding the comparison group selected for extant studies in military samples. With 1 exception [16], studies of suicide exposure based on military samples have compared service members or veterans who knew someone who died by suicide with those who did not. Therefore, the comparison group included individuals who were unexposed to any type of sudden death and those exposed to sudden death from other causes such as natural events (eg, cardiac arrest) or unnatural events (eg, combat and vehicular crashes). An understanding of how a suicide loss differs from other sudden and traumatic losses is needed for the design of postvention interventions [17].

There is also an absence of studies of modifiable moderators that could be targeted to promote healing among those affected by a suicide death. Studies of risk factors following suicide exposure has primarily examined sociodemographic variables, such as family and personal history of psychiatric disorders and suicidal behavior, relationship with the decedent (eg, kinship and perceived closeness), and the number of trauma exposures, none of which are modifiable [7,18]. An additional knowledge gap is that we know almost nothing about help seeking following a suicide death and whether the received services and supports (eg, support groups for suicide death survivors) facilitate healing. The 1 study to examine the effect of suicide exposure on mental health treatment seeking in veterans was based on a small university sample [19].

In summary, the numbers of veterans needing intervention following suicide exposure, the types of problems they are experiencing, and the services and supports that might most benefit them are all unknown.

### This Study

This paper describes the protocol for a US Department of Veterans Affairs (VA) Health Services Research and Development–funded study on suicide exposure among veterans within 6 years of military separation. Veterans who have served on active duty since September 2011 are known as “post-9/11” veterans. The study’s primary objective is to describe the mental health outcomes associated with suicide exposure among post-9/11 veterans within the first 6 years of military separation, when their risk for suicide is elevated [1,2]. Our sampling strategy aims for adequate representation of female and American Indian and Alaska Native veterans to allow for the examination of associations between exposure and outcomes within these groups. Our secondary objective is to elucidate why some veterans develop mental health problems following suicide exposure, whereas others do not. Consistent with the socioecological model of suicide prevention, risk and protective factors are grouped at individual, relational, community, and societal levels [20]. We will include 2 comparison groups—those exposed to sudden death for reasons other than suicide (eg, combat) and those unexposed to any sudden death—to identify the common and unique contribution of suicide exposure to health outcomes and help seeking.

## Methods

### Study Objectives, Design, and Aims

This is a 4-year, explanatory, sequential, mixed methods study of a nationally representative sample of post-9/11 veterans enrolled in VA health care. The study starts with quantitative data collection and analysis (which is the priority of the study), followed by qualitative interviews. Textbox 1 presents the study aims and hypotheses. Primary outcomes comprise mental health problems associated with trauma and loss and suicide precursors (suicidal ideation, attempts, and planning).

**Study aims and hypotheses.**

**Aim 1 (1 of the 2 primary aims of the study)**

- Aim 1 is to evaluate differences in the prevalence of posttraumatic stress disorder; prolonged grief disorder; and suicidal ideation, attempts, and planning among veterans exposed to suicide compared with those exposed to other causes of sudden death and with unexposed veterans. We will also assess whether prevalence differs based on sex and race.

**Hypothesis 1**

- The prevalence of posttraumatic stress disorder; prolonged grief disorder; and suicidal ideation, attempts, and planning will be high in veterans exposed to suicide compared with veterans exposed to other sudden deaths and with unexposed veterans, after controlling for closeness with the decedent and other covariates.

**Aim 2 (1 of the 2 primary aims of the study)**

- Aim 2 is to identify the modifiable moderating factors for the association between suicide exposure and negative outcomes and the modifiable moderating factors for the association between suicide or sudden death exposure and negative outcomes relative to those with neither exposure.

**Hypothesis 2**

- Social support will buffer, whereas stigma will worsen, the effects of suicide exposure on health outcomes after controlling for closeness with the decedent and other covariates.

**Aim 3 (exploratory)**

- Aim 3 is to describe the treatment experiences, reported suicide attempts, and patterns of Department of Veterans Affairs service use among those exposed to a suicide death compared with veterans exposed to other sudden deaths and with unexposed veterans. We will also explore differences based on sex and race.

**Aim 4 (explains and illustrates aim 2 findings among those exposed to suicide)**

- Aim 4 is to contextualize the quantitative findings from aim 2 through interviews with a purposive sample of veterans exposed to suicide. The interviews will focus primarily on modifiable factors at each level of the socioecological model of suicide prevention to better understand the targets for intervention for people affected by a suicide loss.

Primary data collection will be implemented in 3 waves. During wave 1, we will field a brief survey to a stratified random sample drawn from the population of post-9/11 veterans to assess exposure history (suicide, other sudden death, or neither) and exposure characteristics (eg, time since exposure and closeness with the decedent; N=11,400 participants). This will provide prevalence estimates for suicide exposure among post-9/11 veterans. During wave 2, we will survey a stratified random sample of wave-1 participants (4500/11,400, 39.47%) to assess health outcomes, covariates, and moderators. Female and American Indian and Alaska Native veterans will be oversampled in both surveys. During wave 3, we will interview a purposive subsample of 32 participants with a history of suicide exposure to understand how and why potential moderators affect outcomes. We will supplement the surveys and interviews with VA administrative data identifying diagnoses, reported suicide attempts, and health care use.

### Conceptual Framework

**Figure 1:**
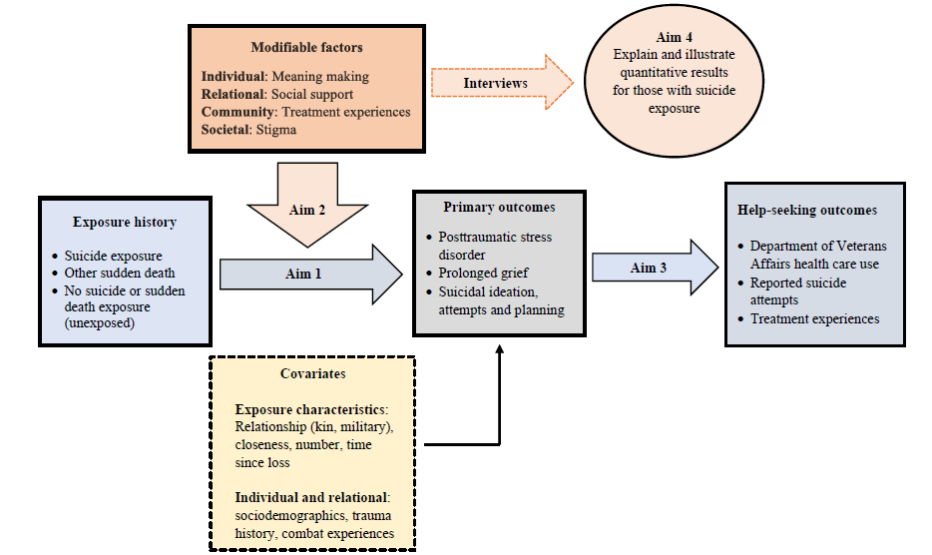
Conceptual model.

Figure 1 presents the study’s conceptual framework. Aim 1 examines differences in outcomes based on exposure history (suicide exposure, other sudden death exposure, or neither), controlling for covariates. Aim 2 examines the moderating effect of modifiable factors, again adjusting for covariates. Aim 3 explores interventions and supports received for identified mental health problems and whether they differ based on exposure history. Aim 4 will help explain and illustrate aim 2 findings for those affected by a suicide loss to further inform interventions.

### Participant Selection and Recruitment and Data Collection

#### Study Population

The study population consists of post-9/11 veterans within 6 years of military separation, who are enrolled in VA medical care.

#### Self-Administered Surveys

The wave-1 survey assesses suicide and other sudden death exposure history and willingness to consider participating in a follow-up web-based survey. We used data from the VA’s data repository, the Corporate Data Warehouse (CDW), to identify the study population, their sex, and their race and to obtain their contact information. The only exclusion criterion is an invalid US mailing address. Veterans in the study population with a valid US mailing address will be stratified based on sex (male or female) and whether they are American Indian or Alaska Native (yes or no).

To allow time for recruitment following sample identification, the recruitment pool includes post-9/11 veterans within 5 years of military separation. As of February 2023, there were 619,216 post-9/11 veterans who were within 5 years of military separation enrolled in VA health care. Of these, 96.82% (599,522/619,216) had valid US mailing addresses. Of those with a valid mailing address, 19.09% (114,424/599,522) were female (gender is inconsistently reported in VA databases and will be assessed as part of this study) and 1.49% (8939/599,522) were classified as American Indian and Alaska Native. We will oversample female and American Indian and Alaska Native veterans, so that female veterans comprise approximately 30% and American Indian and Alaska Native veterans collectively comprise approximately 12% (4% American Indian or Alaska Native female veterans and 8% American Indian or Alaska Native male veterans) of those recruited. The recruitment pool will be updated every 3 to 6 months.

Wave-1 recruitment material will be sent through the US postal service and include a letter that describes the study, a 2-page questionnaire, a postage-paid return envelope, and a pen and note pad as nonconditional incentives. We will append bright-colored Post-it notes to the survey to encourage participation [21]. The notes will be customized for the 4 demographic strata (American Indian or Alaska Native female individuals, American Indian or Alaska Native male individuals, female individuals from other racial groups, and male individuals from other racial groups). The survey coversheet will include a shortened URL and a QR code that recipients can scan if they prefer to complete the survey on the web and a phone number to call if they prefer to complete it over the phone. We will use repeat mailings, emails, and phone calls to encourage participation among nonresponders. The survey can be completed in <10 minutes, as demonstrated by members of the veteran engagement group who provided feedback about the study material. A sample of 11,400 wave-1 participants should provide a sufficiently large pool of veterans in each exposure group for the wave-2 survey. Although we hope to achieve a response rate >50%, response rates to a survey about suicide exposure in this population are not known and will be evaluated over the course of this study.

The wave-2 survey assesses outcomes, covariates, moderators, and experiences of getting help and support for the suicide, sudden death, or another significant loss. Inclusion criteria are responding to the first survey, having a valid email address (provided on the wave-1 survey or identified though CDW), and indicating willingness to consider participating in the wave-1 survey. Those who meet these criteria will be stratified based on exposure history (suicide, sudden death unrelated to suicide, or neither) and, within exposure history group, sex and whether they have been identified as an American Indian or Alaska Native so that we can monitor wave-2 survey participation rates based on these characteristics and evaluate the possibility of adjustment in our sampling plan or recruitment strategy. We plan to randomly select 1500 participants with suicide exposure, 1500 participants with sudden death exposure but no suicide exposure, and 1500 participants with neither suicide exposure nor sudden death exposure from the eligible wave-1 survey respondents to participant in the wave-2 survey. The selection within each arm will match the sex and race composition targets of the wave-1 survey.

We will recruit those who meet the above mentioned inclusion criteria through email. The email will include information about the study, a hyperlink to the study website with pictures and information about the study incentives, and a URL for the web-based survey. Nonresponders will receive reminder emails, a postcard, and outreach calls. Participants will be able to choose an incentive valued at approximately US $20 after completing the survey. On average, members of the veteran engagement group completed the wave-2 survey in 45 minutes. The target sample size for the wave-2 survey is 4500 participants, with the goal of approximately even distribution across the 3 exposure history groups. The feasibility of achieving that goal will depend on the prevalence of suicide and sudden death exposure and response rates.

We will use Qualtrics Federal Risk and Authorization Management Program electronic survey software (Qualtrics XM) for the web-based surveys. Participants will access the website using a secure, shortened URL link and unique personal identification number assigned to them; protected health information will be kept behind the VA firewall. The Qualtrics surveys use branching logic, required fields, and pop-up warnings to reduce missing data. Participants’ survey responses are stored in a web-based database as the participant progresses through the survey.

#### Interviews

We will conduct qualitative interviews with a subsample of at least 32 of the participants who have been exposed to suicide. Veterans who indicated on the first survey that they were at least somewhat close to a person who died by suicide within the past 5 years will be eligible for inclusion. The 5-year criterion will help ensure that participants can remember the help they received after the suicide loss.

Veterans will be purposively selected for interview after quantitative data collection and preliminary analyses [22]. Half of the interview subsample will be veterans with clinically significant symptoms based on our primary outcomes; the other half will have no clinically significant symptoms. Our purposive sampling strategy will ensure a demographically diverse group of participants in terms of sex, race, ethnicity, and relationship (family member, fellow veteran, friend, etc) with the decedent. Half of the sample will be women (as self-reported in the wave 2 survey) and at least half will be from racial and ethnic minority groups, with oversampling of American Indian and Alaska Native veterans.

We will use mail and email to initiate the recruitment of our interview sample. The study team will call veterans within 1 week of sending the recruitment material to discuss participation and will repeat attempts to contact those selected for recruitment. We anticipate contacting approximately 64 of the wave-2 responders who meet our interview inclusion criteria, to achieve the target sample size of 32 interview participants.

We will conduct semistructured interviews with participants over the phone or through an approved videoconference platform. A team member will conduct the interview while another manages the logistics and takes notes. The interview will focus on the modifiable factors included in the study’s conceptual model and measured quantitatively in the second survey. Veterans will also be asked for recommendations that could help other veterans after a suicide loss. Consistent with the explanatory, sequential, mixed methods design, we will use the quantitative findings to inform the content of the qualitative interviews. The semistructured interview guide will be further refined based on feedback from veterans participating in the study’s veteran engagement group and from professional experts in postvention and trauma participating in our advisory panel. Interviews are anticipated to last up to 60 minutes.

### Stakeholder Engagement

This study has 2 stakeholder engagement panels to ensure that the work is meaningful and useful to the target population, build trust in the study findings and results, and accelerate the uptake and translation of the study findings into clinical practice [23,24]. The first is a veteran engagement group with experience in consulting to researchers about research projects. At the time of proposal development, the veteran engagement group consisted of 10 veteran members varying in health status and personal backgrounds, including 5 female individuals, 4 post-9/11 veterans, 5 racial and ethnic minority individuals (1 American Indian or Alaska Native, 2 African American, 1 Pacific Islander, and 1 Asian), and 1 Hispanic or Latinx veteran. The veteran engagement group provided feedback about the topic and recruitment material during grant development. During the project, they will meet with the research team at least 3 times to provide feedback about the recruitment materials, survey content, and dissemination to veterans. The second panel comprises staff with roles in VA’s suicide prevention program, the Mental Illness Research Education and Clinical Center for suicide prevention, and the National Center for PTSD and an expert in prolonged grief disorder (PGD) from outside the VA. This professional advisory panel provided consultation about the topic and its relevance for VA health care, the study-specific risk management protocol, and the survey measures to assess treatment experiences. They will ensure that the study team is aware of relevant policies and initiatives and assist with dissemination to health care system–level stakeholder groups (clinicians, program managers, and operations leaders), as relevant.

### Risk Management

Resources are provided to enhance safety and access to mental health resources for all veterans recruited for participation. For example, the recruitment material and surveys include information (either written instructions or a hyperlink) to access the Veterans Crisis Line. Furthermore, the study team will implement a detailed protocol if participants exhibit concerning behavior or express thoughts of self-directed or other-directed violence in written content on surveys or during phone calls or interviews. This protocol includes administration of the Columbia–Suicide Severity Rating Scale Adult Screener for Primary Care [25] and a warm handoff to the Veterans Crisis Line for a positive screen when possible elevated acute risk is identified.

### Measures

Factors considered in measure selection were reliability and validity, use in related studies (to facilitate comparison across studies), veteran engagement panel feedback about relevance and acceptability, and participant burden. Secondary and exploratory measures are included to develop a more comprehensive understanding of the effects of suicide exposure and inform future studies.

#### Exposure History and Characteristics

Questions assessing suicide exposure history and exposure characteristics were drawn from the Military Suicide Research Consortium recommendations for common data elements and modified for clarity based on recommendations from the study’s veteran engagement group.

*Suicide exposure* will be assessed using the question, “Did you personally know anyone who has died by suicide?”

*Other sudden death exposure* will be assessed using the question, “Did you know anyone who died suddenly from something other than suicide?” The latter question is followed with an explanation, “By ‘suddenly’ we mean the death was not expected or occurred within a few days. Causes of sudden death include natural disaster, medical illness (such as cardiac arrest or stroke), combat, car crashes, accidents or homicide.”

For each exposure type, we will ask 6 questions. Questions 1 and 2 ascertain the number of exposures and year of the most recent exposure. As individuals may have multiple exposures and closeness to the decedent is related to outcomes [26], the remaining 4 questions ask participants to think of the person with whom they were closest who died by suicide or other sudden death while rating closeness to the decedent (5-point Likert scale ranging from “not close at all” to “very close”), perceived impact of the death (5-point Likert scale ranging from “the death had little effect on my life” to “the death had a significant or devastating effect on me that I still feel”), relationship with the decedent (eg, family member or fellow service member), and year of the death of the person with whom they were closest.

#### Primary Outcomes

*PTSD* in the past month will be assessed using the PTSD Checklist version 5, a validated 20-item self-report measure to assess the Diagnostic and Statistical Manual of Mental Disorders–Fifth Edition (DSM-5) symptoms of PTSD, with scores ranging from 0 to 80 [27,28]. Participants will be asked to complete the PTSD Checklist version 5 with respect to “the very stressful experience” that is on their mind and bothers them the most, as assessed using the measure of trauma history described in the following section. At the time of protocol development, a score ≥33 was considered to be indicative of probable PTSD [29].

*PGD* will be assessed using the 10 items from the Prolonged Grief-13–Revised (PG-13–R) that assess DSM-5 Text Revision symptoms of PGD on a 5-point Likert scale, with total scores ranging from 10 to 50 [30]. PG-13–R is a reliable and valid measure of maladaptive grief response consistent with the new diagnosis of PGD. Participants are asked to complete PG-13–R with respect to the “the death that was the most difficult.” Following completion of the measure, participants indicate the cause of the death, year of the death, and relationship with and closeness to the decedent. Those who indicate that the cause of death was suicide or other sudden death will not be prompted to indicate closeness to and relationship with the decedent, as both were assessed in the wave-1 survey. A score ≥30 is indicative of probable PGD [30].

*Suicide ideation*, *attempts*, and *planning* will be assessed using the 4-item Suicidal Behavior Questionnaire–Revised [31], a measure recommended for population-based surveys because of its utility, appropriateness, and psychometric properties [32]. Item 1 assesses lifetime suicide ideation and attempts, item 2 assesses past-year suicidal ideation, item 3 assesses the threat of suicide attempt, and item 4 evaluates the self-reported likelihood of suicidal behavior in the future. We added an item to identify the person to whom participants communicated the threat of suicide attempt (item 3). Response to each item will be used to evaluate the prevalence of lifetime and past-year suicidal ideation, attempts, and planning. We will also compute total scores (range 3-18). Scores >7 are indicative of possible suicide risk in nonclinical samples [31].

#### Secondary Outcomes of Functioning

*Community reintegration* will be assessed using the Military to Civilian Questionnaire, a 16‐item self‐report measure of reintegration difficulty across 5 domains of functioning [33]. Items are rated on a 5-point Likert scale ranging from “no difficulty” to “extreme difficulty.” Total scores range from 0 to 4, with high scores indicating more reintegration difficulty. The scale’s factor structure and association with theoretically related measures support construct validity. We are piloting the inclusion of 2 additional items to assess financial difficulty and housing instability or homelessness.

*Physical and mental health–related quality of life* will be assessed using the Veterans Rand–12 item health survey, an adaptation of the 36-item Medical Outcomes Study health survey that has been extensively validated in outpatient VA populations [34]. Veterans Rand–12 physical component and mental component summary scores are comparable with 36-item versions and standardized to the US population. Scores range from 0 to 100 (mean 50, SD 10).

#### Secondary Clinical Outcomes

*Depression* will be assessed using the 8-item Patient Health Questionnaire, a screening measure to assess the prevalence and severity of depression in epidemiological studies that has strong psychometric properties [35]. Total score ranges from 0 to 24, with scores ≥10 indicative of current depression and scores ≥20 indicative of severe depression.

*Anxiety* will be assessed using the Generalized Anxiety Disorder 7-Item [36,37]. This scale is based on the diagnostic criteria for generalized anxiety disorder described in the Diagnostic and Statistical Manual of Mental Disorders–Fourth Edition, but there is evidence supporting its reliability and validity as a measure of anxiety in the general population [37]. Scores range from 0 to 21, with scores between 10 and 14 indicating moderate anxiety and scores between 15 and 21 indicating severe anxiety. A score ≥10 will be used to identify probable anxiety disorder [36].

#### Exploratory Clinical Outcomes

*Exploratory clinical outcomes* are other known risk factors for suicide among veterans [38].

*Sleep disturbance* and *impairment* will be assessed using the Patient-Reported Outcomes Measurement System Short Form Sleep Disturbance and Sleep-Related Impairment scales. These two 4-item scales (8 items in total) are rated on a 5-point Likert scale and yield total scores that range from 4 to 20 and can be converted to *t* scores, with high scores indicating great sleep disturbance or impairment [39].

*Probable alcohol* and *drug problems* will be assessed using the Two-Item Conjoint Screen [40]. This screen was included in the Department of Defense Post-Deployment Health Reassessments. A cutoff score of 1 had 0.80 sensitivity and specificity in patients in primary care.

*Pain intensity and interference* in the past week will be assessed using the 3-item Pain, Enjoyment of Life, and Activity scale. This scale has been found to have good reliability and construct validity [41]. Participants will also indicate the main causes of their pain (eg, a combat injury or a chronic health problem).

#### Covariates

*Sociodemographics assessed using VA administrative data* include sex, age, race, time since military separation, VA disability ratings, psychiatric diagnoses, medical diagnoses, and zip code of home residence. Medical diagnoses will be used to compute the Charlson Comorbidity Index scores using International Classification of Diseases–10th Revision codes [42,43]. Zip code will be used to determine region and rurality, which is associated with suicide risk [44]. These measures are available for responders and nonresponders and will be used to adjust for response bias.

*Sociodemographics assessed using self-report* include marital status, number of times married, education, employment, sexual orientation, and parental status. We will identify race, ethnicity, and gender identity because these variables are incomplete in administrative data. Those identifying as American Indian or Alaska Native will be asked to specify their nation or tribal affiliation, whether they reside in their reservation homeland, and whether they receive health care through the Indian Health Service. We will assess age of entry into the military, military branch and component, rank, and number of combat deployments. We will use single-item questions to assess mental health treatment history (eg, age of first mental health treatment and receipt over the past 12 months). Those who received mental health care within the past 12 months will be asked to indicate the location of care (VA or non-VA).

*Trauma history* will be assessed using a modified version of the Trauma Screen from the PTSD Diagnostic Scale for DSM-5. Participants indicate the very stressful events that they have experienced or witnessed at any time in their lives from a list of 8 categories (eg, assault, sexual trauma as adult, or child abuse) [45]. A follow-up question asks which of the endorsed “very stressful experiences” is on their mind and bothers them the most.

*Combat experiences* will be assessed using the 13 items designed to capture experiences commonly encountered during Iraq and Afghanistan deployments that are included in the Millennium Cohort Study, the largest population-based, prospective study of health and well-being in US military history. Items will be summed (range 0-26), and combat severity will be categorized as no combat (0 items), low combat (1-3 items), medium combat (4-7 items), and high combat (8-13 items), as done in previous studies [46].

#### Potentially Modifiable Factors

*Social support* will be assessed using the Postdeployment Social Support Scale from the Deployment Risk and Resilience Inventory-2 [47]. This scale measures the extent to which family, friends, coworkers, employers, and community provide emotional and instrumental support. Each of the 10 items is scored on a 5-point Likert scale, with responses ranging from “strongly disagree” to “strongly agree.” Total scores range from 10 to 50. The scale has high internal consistency, and associations with mental health outcomes support its construct validity.

*Perceived stigma* will be assessed using the 10-item version of the stigmatization subscale from the Grief Experience Questionnaire [48]. Items (eg, “feel like the death somehow reflected natively on you or your family”) are rated on a 5-point Likert scale, with responses ranging from “never” to “almost always” and total scores ranging from 10 to 50. Consistent with previous studies [48,49], we modified the stem to elicit reactions following the suicide or sudden death of the closest contact identified in the first survey. Those who have had both suicide and sudden death will be asked to select the death that was most difficult for them. Participants in the unexposed group will not complete this measure.

*Meaning making* will be assessed using the 6-item Integration of Stressful Life Experiences Scale–Short Form [50]. High scores indicate more adaptive meaning made following a loss. The measure has good internal consistency, test-retest reliability, and convergent validity with scales for psychiatric and bereavement distress. A total score ≤20 may be used as a cutoff for indicating problems with meaning made of loss [51]. We modified the stem to elicit reactions following the suicide or sudden death of the closest contact identified in the first survey. Those who have had both suicide and sudden death will be asked to select the death that was most difficult for them. Participants in the unexposed group will not complete this measure.

*Everyday discrimination*, an exploratory measure associated with suicide risk [52], will be assessed using the Everyday Discrimination Scale–Short Version [53]. This scale assesses frequency of exposure to 5 types of discrimination in “day to day life” rated on a 5-point scale (“never” to “at least once a week”). An additional item assesses the main reasons (eg, gender, race, and religion) for discrimination experienced at least “a few times a year.”

#### Help-Seeking Outcomes

*Treatment experiences for the loss or losses* will be assessed using 14 items developed for this study based on the UK survey by Pitman et al [54] for help seeking and support following suicide and other sudden death exposure and recommendations from the study’s advisory panel. Participants indicate whether they received interventions or supports after the loss from a checklist (yes or no) and, if received, how helpful it was to them on a 5-point scale ranging from “not at all helpful” to “extremely helpful.” They are asked to describe other types of assistance or support they wish they had received and whether they have participated in “an event or gathering to increase awareness of suicide and suicide loss.” Participants are instructed to complete this measure with respect to the suicide or sudden death of the person closest to them, as reported in wave 1, or the loss that was most difficulty for them, as identified for PG-13–R. We will derive four binary outcomes from these questions to indicate (1) receipt of any intervention from a medical professional (eg, therapist or physician), (2) receipt of any support from clergy or spiritual leaders, (3) receipt of any informal support or interventions (eg, community members, elders, friends, and family), and (4) receipt of any support or intervention rated as at least “somewhat helpful.”

#### VA Health Care Use

We will extract CDW data about service use for the 12 months before wave-2 survey completion. Service use will be classified as mental health, primary care, and specialty care. We will use procedure codes to classify outpatient mental health care as individual psychotherapy, group psychotherapy, or other. We will use pharmacy data to identify psychiatric medication prescriptions (eg, antidepressant, antipsychotic, lithium salts, prazosin, and benzodiazepines). We will use data from templated notes to identify the use of evidence-based psychotherapies for PTSD, depression, and insomnia among those who meet the survey criteria for these conditions. Outcomes will be binary (yes vs no for each type of service) and continuous (number of appointments per service category and inpatient length of stay over the preceding 12 months).

*Suicide attempts reported to VA clinicians* will be extracted from the CDW, based on data automatically generated from the Comprehensive Suicide Risk Evaluations and Suicide Behavior and Overdose Report, either of which VA requires for reporting suicide behavior. We will identify nonfatal events using the self-directed violence classification of suicide attempt with injury or suicide attempt without injury, based on self-directed violence classification guidelines developed by the Centers for Disease Control and Prevention and adopted by the VA.

### Analysis

#### Power

The anticipated wave-2 sample size (n=4500) will provide >90% power for a global likelihood ratio test and for pairwise comparisons for a range of considered differences between exposure history groups in the distribution of a binary outcome (hypothesis 1). The configurations considered for the trio of outcome rates in these calculations comprised differences between the middle rate and each of the lower and higher rates corresponding to approximately half of the middle rate. Among female veterans (anticipated n=900), the study should have >82% power. Among American Indian and Alaska Native veterans (anticipated n=540), the global comparisons will have >82% power. Power for the pairwise comparisons will be limited for comparisons involving an exposure history group with very low representation of American Indian or Alaska Native veterans. Power to detect moderation (hypothesis 2) is determined by sample size, prevalence of the exposure history classification, prevalence of the moderator within each group, and outcome rates for the different combinations of exposure and moderator measures. Under different plausible scenarios for these measures, power to detect the interaction ranges from 0.81% to 0.87%.

As discussed previously, little is known about the response rates of veterans to surveys regarding suicide exposure. The study population is sufficiently large that obtaining ≥10,000 respondents for the wave-1 survey is highly likely by sufficiently increasing the selected sample for this aim. The precision of the estimation of exposure rates among this population then will be less affected by sample response rates and sample size than response bias. For wave 2, we may find that those with no exposure are less willing to participate and that the cohort of wave-1 respondents willing to participate in wave 2 is shifted toward those with suicide or other sudden death exposure. In this event, we will likely need to alter the planned analysis to focus on comparing the suicide and other sudden death exposure groups. However, we would expect that the power available for the analyses will be comparable with or larger than the power discussed previously for analyses within female and American Indian and Alaska Native veterans.

#### Preliminary Analyses

We will construct nonresponse-adjusted, sample inclusion probability–weighted estimates of the prevalence of suicide exposure, sudden death exposure, and the combination of exposures (exposure to suicide and other sudden death) and the prevalence of the study outcomes. We will use administrative data together with the survey sampling strata to develop logistic regression models for the probability of survey response for each survey. We will then adjust the initial survey weights based on the estimated probability of survey response to generate weights for use in analysis. If there is <3% of missing data for the survey items required for a particular analysis, we will further adjust the weights within classes of individuals with similar response propensities from the same stratum to address missing data. If the amount of missing survey items exceeds 3%, we will use the available survey and administrative data in a chained sequence of regression analyses to impute missing survey items [55]. We will then use the imputed data to implement the weighted analyses described in the following section.

#### Aims 1, 2, and 3

For aim 1, we will use nonresponse-adjusted, sample inclusion probability–weighted logistic regression analyses for the outcomes with sampling strata and exposure categories as explanatory measures. We will conduct global Rao-Scott likelihood ratio tests to assess the differences in prevalence of the primary outcomes between exposure history groups followed by tests for pairwise differences between groups (hypothesis 1). To assess whether associations between exposure and outcomes persist when adjusting for covariates, we will expand these models to include closeness to the decedent or decedents, an interaction between exposure history group and closeness, and the sociodemographic and trauma history covariates. If there does not appear to be an interaction, we will simplify the model. We will also expand the analyses to include interactions of exposure history group with number of death exposures, time since exposure, and time since military separation to examine whether associations vary with these covariates. We will summarize the differences between exposure history groups using model-estimated odds ratios and CIs.

To assess whether the differences between exposure history groups vary with sex and race, we will use similar Rao-Scott likelihood ratio tests to compare weighted logistic regression models incorporating exposure history, sex or race, the respective interaction with exposure, closeness of decedent, and sociodemographic and trauma history covariates with models that do not include the exposure by sex or exposure by race interaction. Additional analyses will examine the differences in total scores (continuous measures) for the outcomes, modifying the abovementioned analyses to use weighted multiple linear regression models. Similar likelihood ratio tests will be used and the differences between exposure history groups will be summarized with model-estimated least square mean differences and corresponding CIs. We will also conduct a similar set of analyses to explore whether the prevalence of the outcomes differ based on the use of VA services (any and mental health) in the previous year.

For aim 2, we will add the potential modifying factor (eg, social support or stigma for hypothesis 2) and the interactions with exposure history and with decedent closeness to the model developed for aim 1. We will use Rao-Scott likelihood ratio tests to assess whether the association between exposure history and a given outcome varies across levels of the moderating factor or across levels of exposure and decedent closeness. If we obtain significant results for a test of an interaction between exposure and a moderator, model-estimated associations between the moderator and the outcome within exposure history groups and additional pairwise contrasts between exposure history groups conditional on the various values of the moderator will be examined.

For our exploratory aim 3, we will create summary measures of treatment experiences, reported suicide attempts, and VA service use and then, similar to aim 1, construct weighted estimates of the prevalence of these treatment measures, along with 95% CIs, in the exposure history groups and compare these using weighted logistic regression analyses with Rao-Scott likelihood ratio tests. Within exposure history groups, among those we have identified as meeting the survey criteria for the clinical conditions, we will estimate the population rates of receiving VA mental health care (yes, no, number of sessions, type of therapy, and report of attempt) during the year before the survey using finite population sampling domain estimation methods. We will then assess whether these vary based on sex and race.

#### Aim 4 Qualitative Analysis

We will use a qualitative data management software package to facilitate the coding and retrieval of qualitative themes. Coders will apply a priori–determined, first-level codes that correspond to the interview domains (eg, social support, professional help, and stigma). We will use a constant comparative analysis approach in which we begin to review data as soon as the first interviews are conducted to permit the identification of emerging issues to be included where appropriate in the ongoing interviews. We will review all proposed codes in weekly meetings to derive an initial codebook. We will elaborate upon the codebook and adjust content as each interview is reviewed, through practice coding and interview reviews until we have finalized the code list. Before independent coding, we will practice coding as a team until we reach consensus and record all decisions to create an audit trail. In addition, we will double code at least 20% of the transcripts, ensuring that each coder codes at least one of the transcripts of each of the other coders and ensure acceptable interrater reliability. Discrepancies will be resolved in team meetings. After coding is complete, we will produce code reports that include all subcodes for each selected first-level code and by veteran mental health status (clinical vs nonclinical interview groups). We will use the code reports to synthesize patterns of responses related our domains of inquiry into larger themes.

For aim 4, interpretation will involve the identification of differences in modifiable factors across our clinical and nonclinical groups. For example, we will examine whether veterans who differ in outcomes describe differences in how people in the community supported them or in their treatment experiences. We will examine convergence and expansion across the quantitative and qualitative data [56].

### Ethical Considerations

The Minneapolis VA Health Care System Insitutional Review Board (IRB) granted this protocol (reference number 1676462) an exempt determination under category 2 (research that involves surveys or interview procedures) subparts 2 (disclosure would not be harmful) and 3 (IRB conducts limited review to make the required determination that there are adequate provisions to protect the privacy of participants and to maintain the confidentiality of data). The IRB also granted a Waiver of Health Insurance Portability and Accountability Act Authorization for all study procedures involving the collection or use of protected health information. The overseeing Research and Development Committee has reviewed and approved all amendments or modifications to study procedures and materials.

## Results

This project began on July 1, 2022. The study team includes experts in suicide exposure, suicide prevention, traumatic stress, epidemiology, survey development and methods, statistics, programming, and VA administrative data. A veteran engagement group and an advisory panel of professional experts provide additional guidance throughout the study. Milestones so far include the identification of the sampling frame, implementation of the first phase of the stratified random sampling plan, refinement and preparation of the recruitment material and surveys, and piloting of survey methods in a sample of approximately 800 veterans to identify and resolve challenges before scaling up. Pilot enrollment began on May 1, 2023.

The study’s funding period will end on June 30, 2026. The veteran engagement group will help us identify the findings that are most important to veterans, including study participants and dissemination vehicles (eg, the study website, publications for service members and veterans, and veterans service officers). We will collaborate with the professional advisory panel to disseminate key findings to VA operational stakeholders and clinicians. Findings will be disseminated to the scientific community through publications and presentations.

## Discussion

### Summary

This is the only national, population-based study of suicide exposure in veterans and the first one designed to study differences based on sex and race. It is an initial, crucial step in addressing the dearth of studies of suicide risk in American Indian and Alaska Native veterans and fills the knowledge gaps identified by the US National Alliance for Suicide Prevention, including the need for reliable and valid estimates of suicide exposure and the impact of exposure to suicide [17]. This information is needed to identify postvention strategies and interventions that address the needs of those affected by suicide loss or combinations of suicide, other losses, and trauma and whether these needs differ based on sex and race.

We are prioritizing outcomes associated with trauma and loss. However, the rich data set this study creates may yield novel information about the effect of suicide exposure on health and functioning that have not been hypothesized. Analyses involving secondary and exploratory measures and VA health care use may pave the way for full understanding of the effects of suicide loss and help identify those at risk for being most affected. As such, we anticipate that this data set will lay the foundation for studies that go beyond the aims of this project. Possibilities for future studies include epidemiologic studies of outcomes beyond those we are able to examine as part of this project (eg, the longitudinal course of physical and mental health effects of suicide loss and other sudden death) and, alternatively, studies that prioritize qualitative data collection and analysis to obtain a more complete understanding of risk and protective factors following exposure to suicide or other sudden and traumatic death.

A challenge for studies in this area is that veterans may have been exposed to >1 suicide or other sudden death from combat or other reasons [9,16]. Consistent with previous studies, we are asking participants to focus on the suicide and sudden death of the person with whom they were closest because this is the loss that is likely to have the most significant effect on them [26]. Relatedly, participants may have PTSD owing to the suicide exposure or another stressor, and they may have PGD owing to suicide exposure or another death. To provide more clarity regarding the sources of their symptoms, we are asking participants to specify and use as a referent the trauma and death that was the most difficult for them. However, participants may have difficulty in separating their response to 1 loss or trauma from their responses to others. In our analyses, we will examine the timing, number, and combinations of exposures in an effort to better understand how multiple losses and trauma of different types interact to affect outcomes.

We are assessing exposure history through survey because we are not able to ascertain exposure history through medical records. Challenges associated with survey data collection include the possibility that response rates may be lower than expected and vary based on exposure history, sex, or race. In anticipation of these potential problems, we will recruit in batches, closely monitor participation rates, and adjust the recruitment methods and the sampling plan in an effort to approximately achieve the desired number of wave-1 and wave-2 responders per stratum. In the analysis, we will use inverse probability weights to help account for differential responses and weigh estimates back to the population. We will also report all results with CIs as measures of precision, irrespective of *P* values, so that our results can still lay a foundation for future studies.

We also recognize that the prevalence of suicide exposure, other sudden death exposure, and neither may be different than expected based on previous studies with veterans from earlier service eras [12]. If the wave-1 survey does not provide an adequate number of veterans in each exposure history group for our second survey, we may have to adjust our analysis plan. This may occur if, for example, the proportion of post-9/11 veterans unexposed to either suicide or sudden death is very low.

This study does not allow us to examine the longitudinal course of symptoms in our exposure history groups, an important topic with clinical implications. Unfortunately, additional waves of data collection would not be feasible within the funding mechanism’s budget cap. We will, however, be able to evaluate the effect of time since exposure in our analyses. A limitation associated with our sampling strategy is the lack of inclusion of post-9/11 veterans who are not enrolled in VA health care and are at high risk for suicide [1]. However, as not all enrolled veterans use service regularly, we will be able to compare those who have with those who have not used VA health care in the previous year.

Despite these limitations, this study addresses knowledge gaps and will direct VA and the field toward an understanding of the most critical outcomes among veterans exposed to suicide and the mechanisms that may lead to deleterious outcomes and lay a foundation for the development and implementation of effective postvention interventions.

### Conclusions

This study addresses an understudied risk factor for suicide—suicide exposure. Integrating survey, VA administrative, and qualitative data to address significant knowledge gaps regarding the effects of suicide exposure will lay the foundation for interventions to address the needs of post-9/11 veterans affected by a suicide death, including female and American Indian and Alaska Native veterans. By advancing the scientific understanding of suicide loss compared with other sudden deaths, findings have the potential to also inform postvention research and interventions more broadly.

## Appendix

Multimedia Appendix 1 Peer review of grant material from the HSR-4 Mental and Behavioral Health, Health Services Research Parent IRG, Office of Research & Development, HSR4 (USA).

## Abbreviations

CDW: Corporate Data Warehouse
DSM-5: Diagnostic and Statistical Manual of Mental Disorders–Fifth Edition
IRB: institutional review board
PG-13–R: Prolonged Grief-13–Revised
PGD: prolonged grief disorder
PTSD: posttraumatic stress disorder
VA: Department of Veterans Affairs

## References

[1] U.S. Department of Veterans Affairs (VA), Office of Mental Health and Suicide Prevention (2019). 2020 national veteran suicide prevention annual report. https://www.mentalhealth.va.gov/docs/data-sheets/2020/2020-National-Veteran-Suicide-Prevention-Annual-Report-11-2020-508.pdf.

[2] Shen YC, Cunha JM, Williams TV (2016). Time-varying associations of suicide with deployments, mental health conditions, and stressful life events among current and former US military personnel: a retrospective multivariate analysis. Lancet Psychiatry.

[3] Ravindran C, Morley SW, Stephens BM, Stanley IH, Reger MA (2020). Association of suicide risk with transition to civilian life among US military service members. JAMA Netw Open.

[4] Berman AL (2011). Estimating the population of survivors of suicide: seeking an evidence base. Suicide Life Threat Behav.

[5] Cerel J, Brown MM, Maple M, Singleton M, van de Venne J, Moore M, Flaherty C (2019). How many people are exposed to suicide? Not six. Suicide Life Threat Behav.

[6] Pitman A, Osborn D, King M, Erlangsen A (2014). Effects of suicide bereavement on mental health and suicide risk. Lancet Psychiatry.

[7] Erlangsen A, Runeson B, Bolton JM, Wilcox HC, Forman JL, Krogh J, Shear MK, Nordentoft M, Conwell Y (2017). Association between spousal suicide and mental, physical, and social health outcomes: a longitudinal and nationwide register-based study. JAMA Psychiatry.

[8] Hill NT, Robinson J, Pirkis J, Andriessen K, Krysinska K, Payne A, Boland A, Clarke A, Milner A, Witt K, Krohn S, Lampit A (2020). Association of suicidal behavior with exposure to suicide and suicide attempt: a systematic review and multilevel meta-analysis. PLoS Med.

[9] Pitman AL, Osborn DP, Rantell K, King MB (2016). Bereavement by suicide as a risk factor for suicide attempt: a cross-sectional national UK-wide study of 3432 young bereaved adults. BMJ Open.

[10] U.S. Department of Veterans Affairs (VA), Office of Mental Health and Suicide Prevention (2018). National strategy for preventing veteran suicide 2018-2028. https://www.mentalhealth.va.gov/suicide_prevention/docs/Office-of-Mental-Health-and-Suicide-Prevention-National-Strategy-for-Preventing-Veterans-Suicide.pdf.

[11] Peterson A, Bozzay M, Bender A, Monahan M, Chen J (2022). Those left behind: a scoping review of the effects of suicide exposure on veterans, service members, and military families. Death Stud.

[12] Cerel J, van de Venne JG, Moore MM, Maple MJ, Flaherty C, Brown MM (2015). Veteran exposure to suicide: prevalence and correlates. J Affect Disord.

[13] Bryan CJ, Cerel J, Bryan AO (2017). Exposure to suicide is associated with increased risk for suicidal thoughts and behaviors among National Guard military personnel. Compr Psychiatry.

[14] O'Keefe VM, Reger GM (2017). Suicide among American Indian/Alaska Native military service members and veterans. Psychol Serv.

[15] Stone DM, Jones CM, Mack KA (2021). Changes in suicide rates - United States, 2018-2019. MMWR Morb Mortal Wkly Rep.

[16] Lubens P, Silver RC (2019). U.S. combat veterans' responses to suicide and combat deaths: a mixed-methods study. Soc Sci Med.

[17] National Action Alliance for Suicide Prevention, Survivors of Suicide Loss Task Force (2015). Responding to grief, trauma, and distress after a suicide: US national guidelines: survivors of suicide loss task force. https://theactionalliance.org/sites/default/files/inline-files/NationalGuidelines.pdf.

[18] Feigelman W, Cerel J, McIntosh JL, Brent D, Gutin N (2018). Suicide exposures and bereavement among American adults: evidence from the 2016 General Social Survey. J Affect Disord.

[19] Chen JI, Bozzay ML, Monahan MF, Gryglewicz K, Romero G, Steding LH, Gleason LL, Karver MS (2019). Life after loss: comparing student service member/veteran and civilian mental health characteristics among individuals exposed to death by suicide. J Am Coll Health.

[20] Cramer RJ, Kapusta ND (2017). A social-ecological framework of theory, assessment, and prevention of suicide. Front Psychol.

[21] Garner R (2005). Post-It® note persuasion: a sticky influence. J Consum Psychol.

[22] Palinkas LA, Horwitz SM, Green CA, Wisdom JP, Duan N, Hoagwood K (2015). Purposeful sampling for qualitative data collection and analysis in mixed method implementation research. Adm Policy Ment Health.

[23] Knight SJ, Haibach JP, Hamilton AB, Whittle J, Ono SS, Butler J, Flower M, Ray CD, Pugh MJ, Zickmund SL (2022). Veteran engagement in health services research: a conceptual model. J Gen Intern Med.

[24] Haibach J, Hoerster K, Dorflinger L, McAndrew LM, Cassidy DG, Goodrich DE, Bormann JE, Lowery J, Asch SM, Raffa SD, Moin T, Peterson AL, Goldstein MG, Neal-Walden T, Talcott GW, Hunter CL, Knight SJ (2021). Research translation for military and veteran health: research, practice, policy. Transl Behav Med.

[25] Posner K, Brown GK, Stanley B, Brent DA, Yershova KV, Oquendo MA, Currier GW, Melvin GA, Greenhill L, Shen S, Mann JJ (2011). The Columbia-Suicide Severity Rating Scale: initial validity and internal consistency findings from three multisite studies with adolescents and adults. Am J Psychiatry.

[26] Cerel J, Maple M, van de Venne J, Brown M, Moore M, Flaherty C (2017). Suicide exposure in the population: perceptions of impact and closeness. Suicide Life Threat Behav.

[27] Blevins CA, Weathers FW, Davis MT, Witte TK, Domino JL (2015). The posttraumatic stress disorder checklist for DSM-5 (PCL-5): development and initial psychometric evaluation. J Trauma Stress.

[28] Wortmann JH, Jordan AH, Weathers FW, Resick PA, Dondanville KA, Hall-Clark B, Foa EB, Young-McCaughan S, Yarvis JS, Hembree EA, Mintz J, Peterson AL, Litz BT (2016). Psychometric analysis of the PTSD Checklist-5 (PCL-5) among treatment-seeking military service members. Psychol Assess.

[29] Bovin MJ, Marx BP, Weathers FW, Gallagher MW, Rodriguez P, Schnurr PP, Keane TM (2016). Psychometric properties of the PTSD Checklist for Diagnostic and Statistical Manual of Mental Disorders-Fifth Edition (PCL-5) in veterans. Psychol Assess.

[30] Prigerson HG, Boelen PA, Xu J, Smith KV, Maciejewski PK (2021). Validation of the new DSM-5-TR criteria for prolonged grief disorder and the PG-13-Revised (PG-13-R) scale. World Psychiatry.

[31] Osman A, Bagge CL, Gutierrez PM, Konick LC, Kopper BA, Barrios FX (2001). The Suicidal Behaviors Questionnaire-Revised (SBQ-R): validation with clinical and nonclinical samples. Assessment.

[32] Batterham PJ, Ftanou M, Pirkis J, Brewer JL, Mackinnon AJ, Beautrais A, Fairweather-Schmidt AK, Christensen H (2015). A systematic review and evaluation of measures for suicidal ideation and behaviors in population-based research. Psychol Assess.

[33] Sayer NA, Frazier P, Orazem RJ, Murdoch M, Gravely A, Carlson KF, Hintz S, Noorbaloochi S (2011). Military to civilian questionnaire: a measure of postdeployment community reintegration difficulty among veterans using department of veterans affairs medical care. J Trauma Stress.

[34] Kazis LE, Miller DR, Clark J, Skinner K, Lee A, Rogers W, Spiro A 3rd, Payne S, Fincke G, Selim A, Linzer M (1998). Health-related quality of life in patients served by the department of veterans affairs: results from the veterans health study. Arch Intern Med.

[35] Kroenke K, Strine TW, Spitzer RL, Williams JB, Berry JT, Mokdad AH (2009). The PHQ-8 as a measure of current depression in the general population. J Affect Disord.

[36] Spitzer RL, Kroenke K, Williams JB, Löwe B (2006). A brief measure for assessing generalized anxiety disorder: the GAD-7. Arch Intern Med.

[37] Löwe B, Decker O, Müller S, Brähler E, Schellberg D, Herzog W, Herzberg PY (2008). Validation and standardization of the Generalized Anxiety Disorder Screener (GAD-7) in the general population. Med Care.

[38] McCarthy JF, Bossarte RM, Katz IR, Thompson C, Kemp J, Hannemann CM, Nielson C, Schoenbaum M (2015). Predictive modeling and concentration of the risk of suicide: implications for preventive interventions in the US Department of Veterans Affairs. Am J Public Health.

[39] Yu L, Buysse DJ, Germain A, Moul DE, Stover A, Dodds N, Johnston KL, Pilkonis PA (2011). Development of short forms from the PROMIS™ sleep disturbance and Sleep-Related Impairment item banks. Behav Sleep Med.

[40] Brown RL, Leonard T, Saunders LA, Papasouliotis O (2001). A two-item conjoint screen for alcohol and other drug problems. J Am Board Fam Pract.

[41] Krebs EE, Lorenz KA, Bair MJ, Damush TM, Wu J, Sutherland JM, Asch SM, Kroenke K (2009). Development and initial validation of the PEG, a three-item scale assessing pain intensity and interference. J Gen Intern Med.

[42] Quan H, Sundararajan V, Halfon P, Fong A, Burnand B, Luthi JC, Saunders LD, Beck CA, Feasby TE, Ghali WA (2005). Coding algorithms for defining comorbidities in ICD-9-CM and ICD-10 administrative data. Med Care.

[43] Charlson ME, Pompei P, Ales KL, MacKenzie C (1987). A new method of classifying prognostic comorbidity in longitudinal studies: development and validation. J Chronic Dis.

[44] McCarthy JF, Blow FC, Ignacio RV, Ilgen MA, Austin KL, Valenstein M (2012). Suicide among patients in the veterans affairs health system: rural-urban differences in rates, risks, and methods. Am J Public Health.

[45] Foa EB, Cashman L, Jaycox L, Perry K (1997). The validation of a self-report measure of posttraumatic stress disorder: the Posttraumatic Diagnostic Scale. Psychol Assess.

[46] LeardMann CA, Matsuno RK, Boyko EJ, Powell TM, Reger MA, Hoge CW, Millennium Cohort Study (2021). Association of combat experiences with suicide attempts among active-duty US service members. JAMA Netw Open.

[47] Vogt DS, Smith BN, King DW, King LA, Knight J, Vasterling JJ (2013). Deployment risk and resilience inventory-2 (DRRI-2): an updated tool for assessing psychosocial risk and resilience factors among service members and veterans. J Trauma Stress.

[48] Bailley SE, Dunham K, Kral MJ (2000). Factor structure of the Grief Experience Questionnaire (GEQ). Death Stud.

[49] Pitman AL, Osborn DP, Rantell K, King MB (2016). The stigma perceived by people bereaved by suicide and other sudden deaths: a cross-sectional UK study of 3432 bereaved adults. J Psychosom Res.

[50] Holland JM, Currier JM, Neimeyer RA (2014). Validation of the integration of stressful life experiences scale-short form in a bereaved sample. Death Stud.

[51] Holland JM, Neimeyer RA (2016). Integration of stressful life experiences scale (ISLES). Techniques of Grief Therapy: Assessment and Intervention.

[52] Lee YH, Liu Z, Fatori D, Bauermeister JR, Luh RA, Clark CR, Bauermeister S, Brunoni AR, Smoller JW (2022). Association of everyday discrimination with depressive symptoms and suicidal ideation during the COVID-19 pandemic in the All of US Research Program. JAMA Psychiatry.

[53] Sternthal MJ, Slopen N, Williams DR (2011). Racial disparities in health: how much does stress really matter?. Du Bois Rev.

[54] Pitman AL, Rantell K, Moran P, Sireling L, Marston L, King M, Osborn D (2017). Support received after bereavement by suicide and other sudden deaths: a cross-sectional UK study of 3432 young bereaved adults. BMJ Open.

[55] Raghunathan TE, Lepkowski JM, Van Hoewyk J, Solenberger P (2001). A multivariate technique for multiply imputing missing values using a sequence of regression models. Surv Methodol.

[56] Fetters MD, Curry LA, Creswell JW (2013). Achieving integration in mixed methods designs-principles and practices. Health Serv Res.

