# Supplementary material for: The Effects of Suicide Exposure on Mental Health Outcomes Among Post-9/11 Veterans: Protocol for an Explanatory, Sequential, Mixed Methods Study
Source: JMIR Res Protoc. 2023 Sep 26;12:e51324. doi: 10.2196/51324 (PMC10565621; doi:10.2196/51324)
Supplement: Multimedia Appendix 1 [file resprot_v12i1e51324_app1.pdf]

SUMMARY STATEMENT

PROGRAM CONTACT: **(Privileged Communication)** ***Release Date:*** **09/29/2021**

Revised Date:

Application Number: **1 I01 HX003438-01A1**

Principal Investigator ***Formerly:*** **1I01HX003438-01**

SAYER, NINA A.

Applicant Organization: MINNEAPOLIS VA MEDICAL CENTER

Review Group: **HSR4**
HSR-4 Mental and Behavioral Health

Meeting Date: **08/26/2021** ***RFA/PA:*** **HX21-005**
Council: **OCT 2021** ***PCC:*** **HXOBRI**
Requested Start: **01/01/2022**

Project Title: **Exposure to Suicide Among Post 9/11 Veterans: Prevalence, Correlates and Treatment Needs**
SRG Action: **Impact Score:131 Percentile:8.3 +**

Human Subjects: **30-Human subjects involved - Certified, no SRG concerns**
Animal Subjects: **10-No live vertebrate animals involved for competing appl.**
Gender: **1A-Both genders, scientifically acceptable**
Minority: **1A-Minorities and non-minorities, scientifically acceptable**
Age: **1A-Children, Adults, Older Adults, scientifically acceptable**
Clinical Research - not NIH-defined Phase III Trial

Project Year	Direct Costs Requested
1	262,415
2	455,806
3	273,813
4	280,188
TOTAL	1,272,222

ADMINISTRATIVE BUDGET NOTE: The budget shown is the requested budget and has not been adjusted to reflect any recommendations made by reviewers. If an award is planned, the costs will be calculated by VA Office of Research and Development (ORD) staff based on the recommendations outlined in the BUDGET COMMENT section and any relevant ORD service-specific limitations.

KEY SUMMARY POINTS:

1. **Study fills a gap by taking a national perspective on impact of suicide exposure; oversampling of women, Alaskan natives, and native Americans is a strength; well written; outstanding team and strong advisory board**
2. **A minor concern that reliability of classification of suicide attempts is not well described**

DESCRIPTION (provided by applicant):

Background: The toll of suicide goes way beyond the death of an individual. Those exposed (defined as knowing the person) to a suicide death are at elevated risk for mental illness, physical disorders, impaired social functioning, and fatal and nonfatal suicide behavior. Conclusions from the handful of studies that have examined this topic among Veterans are limited by distinct samples, limited sample sizes and other methodological issues. Additionally, the numbers of women and minority race/ethnicity service members and Veterans in these studies were too small to examine what are likely critical differences by sex and race. Significance: This study will provide foundational information on an understudied risk factor for suicide in a cohort of Veterans at increased suicide risk – post 9/11 Veterans within six years following military separation. It will provide information to inform suicide postvention strategies that target the population of suicide bereaved post-9/11 Veterans as well as those targeting women and American Indian/Alaskan Native (AI/AN) Veterans. Innovation and Impact: (1) Use of VA data to provide the most reliable prevalence estimates for suicide exposure among Veterans to date; (2) Oversampling of vulnerable but understudied populations -- Women and AI/AN Veterans; (3) inclusion of two comparison groups to elucidate the common and unique contribution of suicide exposure to health outcomes and patterns of VA service utilization; and (4) Assessment of the formal and informal supports Veterans receive for mental health problems associated with suicide using both survey and VA healthcare utilization data. Specific Aims: (1) Evaluate differences in the prevalence of posttraumatic stress disorder (PTSD), prolonged grief disorder (PGD), and in suicidal ideation, attempts and planning among Veterans exposed to suicide compared with those exposed to other causes of sudden death and with unexposed Veterans. We will also evaluate differences by sex and race. (2) Identify modifiable moderating factors for the association between suicide exposure and negative outcomes and modifiable moderating factors for the association between suicide or sudden death exposure and negative outcomes relative to those with neither exposure. (3) Describe treatment experiences, interests, reported suicide attempts, and patterns of VA service utilization among those exposed to a suicide death compared to Veterans exposed to other sudden deaths and to unexposed Veterans. (4) Contextualize quantitative findings through interviews with a purposive sample of Veterans exposed to suicide. The interviews will focus on modifiable factors at each level of the socio-ecological model of suicide prevention to better understand targets for intervention. Methodology: This explanatory sequential mixed methods study examines outcomes associated with suicide exposure in a nationally representative sample of post-9/11 Veterans enrolled in VA healthcare. We will collect data in three waves. Wave 1 will implement a national population probability sample using a brief survey to assess exposure history (suicide, other sudden death, neither) and exposure characteristics (e.g., time since exposure) among 11,400 Veteran respondents. Wave 2 will survey Wave 1 respondents, stratified by exposure history (suicide, sudden death, neither), to assess outcomes and variables of interest among 4,500 Veterans (1,500 respondents per exposure group). Wave 3 involves interviews with a purposive subsample of 32 Waves 2 survey responders who have been exposed to suicide but differ in outcomes. Quantitative analysis is the priority of the study; the qualitative component will contextualize the quantitative findings. Next Steps/ Implementation: This work will direct VA and the field towards an understanding of the most critical outcomes among

Veterans exposed to suicide, the mechanisms that may lead to deleterious outcomes, and lay a foundation for understanding the effective treatments and supports needed for Veterans who experience a suicide loss, including women and AI/AN Veterans.

CRITIQUE 1

Exposure to suicide is a significant public health problem. A recent study found that almost half of the US population had been exposed to suicide and among those exposed, over one-third experienced significant distress. Those affected by suicide exposure are at increased risk for psychiatric disorders, impaired social functioning, cirrhosis and sleep disorders, disordered grief, suicide attempts and suicide death. Research on suicide exposure among service members and Veterans is limited to only a handful of studies. One study with Iraq and Afghanistan Veterans showed that 58% of survey responders knew a post-9/11 Veteran who had died by suicide. A study of Veterans in Kentucky found that suicide exposure was associated with depression, anxiety and suicidal ideation and another study of service members and Veterans reported that closeness with the decedent predicted future suicide attempts. Conclusions from these studies are limited because of geographically-limited or nonrepresented samples and there is limited evidence on modifiable risk and protective factors that affect outcomes after exposure to suicide. Furthermore, these studies lack a sufficient number of women and Veterans from diverse racial/ethnic backgrounds to examine differences by sex and race.

The present study addresses these shortcomings through four aims: (1) Evaluate differences in the prevalence of PTSD, PGD, and in suicidal ideation, attempts and planning among Veterans exposed to suicide compared with those exposed to other causes of sudden death and with unexposed Veterans. Assess whether prevalence differs by sex and race. (2) Identify modifiable moderating factors for the association between suicide exposure and negative outcomes and modifiable moderating factors for the association between suicide or sudden death exposure and negative outcomes relative to those with neither exposure. Exploratory aims are: (3) Describe treatment experiences, interests, reported suicide attempts, and patterns of VA service utilization among those exposed to a suicide death compared to Veterans exposed to other sudden deaths and to unexposed Veterans. Differences by sex and race will also be explored. (4) Elucidate quantitative findings through interviews with a purposive sample of Veterans exposed to suicide who vary in mental health outcomes. The interviews will focus on modifiable factors at each level of the socio-ecological model of suicide prevention to better understand targets for intervention.

This study is consistent with HSR&D priority areas of suicide prevention, mental health (including PTSD and Prolonged Grief Disorder) and women's health.

There are currently no similar studies of suicide bereavement, suicide exposure or suicide survivorship in the VA HSR&D's portfolio, QUERI's portfolio, or in clinicaltrials.gov for suicide bereavement or survivorship.

2. Innovation and Impact.

The proposed study will significantly advance the current knowledge regarding the impact of suicide exposure on Veterans. Moderately high innovative aspects of the application include:

1. Use of VA national data for a population-based study of suicide exposure among post-9/11 Veterans enrolled in VHA.
2. Oversampling of women and American Indian/Alaskan Native (AI/AN) Veterans to allow for the examination of gender and race on suicide exposure.
3. Inclusion of two comparison groups to identify the common and unique contributions of suicide exposure to health outcomes and patterns of VA service utilization.
4. Assessment of the formal and informal supports Veterans receive for mental health problems associated with suicide using both survey and VA healthcare utilization data.

3. Approach.

Strengths:

The overall approach is well developed. The study hypotheses are clearly written and the planned analyses correspond to the hypotheses.

The study is guided by a socio-ecological conceptual framework that considers risk and protective factors at the individual, relational, community, and societal levels.

Uses a nationally representative sample of post-9/11 Veterans within 6 years of military separation (which is high risk timeframe for suicide).

Uses a two-stage, stratified sampling plan (30% women and 12% AI/AN) for the initial wave of data collection.

Uses an explanatory, sequential mixed methods design.

Uses an evidence-based multi-model approach to optimize recruitment rates.

Uses a standardized combat experiences survey.

Measures history of suicide attempts using a self-report, validated questionnaire (Suicide Behaviors Questionnaire-Revised) and suicide attempts reported by VHA clinicians and identified in the Corporate Data Warehouse based on responses to the Comprehensive Suicide Risk Evaluations and the Suicide Behavior and Opioid Reports.

Minor Weaknesses:

Procedures for evaluating the reliability of the classification of VHA provider-reported suicide attempts were not described.

The justification for examining prevalence of self-reported suicide attempts and provider-reported suicide attempts, alone or in combination, is unclear.

4. Feasibility (including Sampling, Project Timeline and Staffing).

The sampling strategy is well-described and clearly justified.

The study appears to be adequately powered to test the primary hypotheses that examines pair-wise differences among exposure groups. Additional power calculations were presented for subgroups for Aim 1 and for moderator variables for Aim 2. However, Table 3 did not display power calculations for AI/AN group and were difficult to interpret.

Investigators expect a 60% survey response rate based on prior research conducted by the research team. The study proposes a reasonable action plan if response rates are lower than the anticipated 60% rate; there are specific plans to increase the number of Wave 1 participants if the expected percentages of respondents are not obtained for those with suicide exposure, those with sudden death exposure other than suicide and those with neither type of exposures.

Completion of Project Timeline activities and milestones seem reasonable and feasible.

5. Implementation.

A study Advisory Panel will be constituted with members who represent the Office of Mental Health and Suicide Prevention (Matthew Miller, PhD, Director, Suicide Prevention), National Center for PTSD (Craig Rosen, PhD), and Rocky Mountain MIRECC (Suzanne McGarity, PhD and Georgia Gerard, LICSW), the Minneapolis VAHCS Suicide Prevention Program Manager and Coordinator (David Holewinski, LICSW CBIS and Eric Wittenberg, LICSW, respectively) and expert in evidence-based treatment of PGD (Katherine Shear, MD). The Advisory Panel will provide guidance to help with dissemination by identifying early and final results of particular importance, dissemination targets, and vehicles for dissemination. The identification of modifiable risk factors associated with negative health outcomes for Veterans who have been exposed to suicide has the potential to inform mental health policy and delivery of clinical interventions.

6. Investigator Qualifications.

The research team is outstanding and well-qualified to conduct the study. Dr. Nina A. Sayer (PI) is Deputy Director of the Center for Chronic Disease Outcomes Research (CCDOR; a HSR&D Center of Innovation [COIN]), and Professor of Medicine and Psychiatry in the Departments of Medicine and Psychiatry at the University of Minnesota. Dr. Julie Cerel (Co-I) is a leader in the field of suicide exposure/bereavement and suicide prevention. Dr. Alan Teo has been added as a co-investigator team. Dr. Teo is health services researcher with the Center to Improve Veteran Involvement in Care (CIVIC) at the VA Portland Health Care System with expertise in social support interventions and suicide prevention.

7. Multiple PI Leadership Plan.

Not applicable.

8. Facilities and Resources.

The proposed research will primarily be conducted within CCDOR, a VA Health Services Research &
Development Center of Innovation (COIN) located within the Minneapolis VA Health Care System and have excellent resources and research support to carry out the study aims.

9. Adequacy of Response to Previous Feedback Regarding the Proposed Study.

The revised application was highly responsive to the concerns raised by the reviewers, including two major design changes. 1. The revised application now includes two comparison groups in addition to the suicide exposure group. 2. The suicide outcomes have been improved by extracting provider-reported suicide attempts from the VHA medical record. The description and rationale for the study procedures are also more clearly written.

10. Protection of Human Subjects from Research Risk.

No concerns.

11. Inclusion of Women and Minorities in Research.

No concerns.

12. Budget and Period of Support.

No comment.

13. Sharing Research Data (Data Management and Access Plan (DMAP).

No concerns.

14. Overall Impression.

This project addresses a significant public health problem related to our understanding of the prevalence and the risk and protective factors associated with negative health outcomes (PTSD, Prolonged Grief Disorder, suicidal ideation and suicide attempts) following exposure to suicide for post-911 Veterans in VHA. The revised application was highly responsive to the reviewers' concerns that resulted in improved hypotheses and study design changes. The overall approach is well designed with only a few minor weaknesses.

15. Key Strengths.

1. Well-designed study based on a clear conceptual framework and clearly written hypotheses.

2. Addresses a major gap in our understanding of the role of exposure to suicide among post-911 Veterans.

3. Strong research team that is well-suited to carry out the study aims.

4. Uses an explanatory, sequential mixed methods design.

5. Includes a detailed action plan if the expected sample sizes are not obtained.

16. Key Weaknesses.

1. Evaluation of self-report/provider-reported suicide attempts.

CRITIQUE 2

1. Significance.

Previous research suggests that exposure to suicide death of a close friend or relative is associated with negative outcomes such as PTSD and suicide attempt in the person exposed; however, little is known about the magnitude of the problem among Veterans and especially among women Veterans and Native American Veterans. While the number of Veterans exposed to suicide death is large, little is known about the magnitude of risk for negative outcome compared to those exposed to other sudden deaths.

The revised design of the study will allow disentangling other sudden loss from suicide loss – an important contribution to advancing research in this area.

2. Innovation and Impact.

This will be the first population-based study of suicide exposure in Veterans and the only one designed to study differences by sex and race.

3. Approach.

Strengths
- Mixed methods design.
- Inclusion of 2 control groups.
- Large sample.
- Oversampling hypothesized high risk groups.
- Survey wave design.
- Power analysis section is now clear.

4. Feasibility (including Sampling, Project Timeline and Staffing).

The feasibility and timeline appear to be appropriate.

5. Implementation.

The investigators have an adequate plan and have formed an advisory panel.

6. Investigator Qualifications.

The study team is strong and includes expertise and experience across all the required domains.

7. Multiple PI Leadership Plan.

Not applicable.

8. Facilities and Resources.

No concerns.

9. Adequacy of Response to Previous Feedback Regarding the Proposed Study.

The team has been highly responsive.

10. Protection of Human Subjects from Research Risk.

No concerns.

11. Inclusion of Women and Minorities in Research.

No concerns.

12. Budget and Period of Support.

No concerns.

13. Sharing Research Data (Data Management and Access Plan (DMAP).

No concerns.

14. Overall Impression.

Post-exposure to suicide is under-studied as a risk factor for suicide attempt in the VA. A better understanding of this risk, especially as compared to exposure to other sudden deaths and no exposure might lead to more tailored interventions for suicide prevention (e.g., timely outreach to friends of those whose die by suicide). The application is highly responsive to previous reviews and adequately addresses these critiques. The inclusion of a second control group is a particular strength.

15. Key Strengths.

1. Under-studied area.

2. Inclusion of 2 control groups – exposed to other sudden loss and not exposed to sudden loss.

16. Key Weaknesses.

1. None noted.

CRITIQUE 3

1. Significance.

Inadequate research has been conducted on the influences of death by suicide of a relative/friend on negative mental health outcomes, including exposed-individual PTSD symptoms and suicide risk. This is a particularly relevant problem for Veterans, women Veterans, and Native American Veterans. Without understanding the down-stream effects of suicidal behavior on others, the VA lacks guidance on how to appropriately intervene with suicide-exposed individuals. The aims of the project include evaluating negative mental health outcomes and suicide risk in suicide-exposed Veterans versus those not exposed to sudden loss or exposed to non-suicide related

loss, identifying modifiable moderating factors (e.g., social support, meaning making) that impact negative outcomes, and describing treatment experiences and patterns of VA service utilization. The research focuses on post-9/11 Veterans, which is a group likely to be highly impacted by suicide-exposure. The proposed focus of the project has high clinical importance with potential to inform key clinical HSR&D priority issues including: suicide risk, PTSD, prolonged grief disorder, and women's health.

Weaknesses: None noted.

2. Innovation and Impact.

The proposed research involves an intensive exploration of the impact of suicide-exposure to ongoing Veteran mental health concerns and is innovative in several ways. The research will help to establish valid and reliable estimates of the number of people exposed (Wave 1) and the impact of exposure to suicide, a key priority of the National Action Alliance for Suicide Prevention. The project will utilize Wave 1 data to identify the relevant sample for additional surveys (Wave 2), and it will include two comparison groups to distinguish between suicide exposure and exposure to other death-related experiences or no exposure to sudden loss. It will also oversample for women and Native American Veterans, providing important information on understudied groups. Wave 3 will conduct qualitative interviews with suicide-exposed Veterans to determine more specifics about the psychological impact of suicide exposure. The study has potential to impact and improve current standards of care, of which there is minimal guidance on how to help suicide-exposed Veterans cope with such loss.

Weaknesses: None noted.

3. Approach.

The approach will involve three waves of data collection. Appropriate sampling procedures are specified for identification of suicide-exposed Veterans and two control groups (sudden death exposed and no death exposure). Inclusion and exclusion criteria and data sources are appropriately identified across waves. Appropriate plans for ensuring specified participant recruitment and potential barriers are described, as are key analytic issues such as addressing missing data. Wave 3 of the study includes a qualitative interview that will be transcribed and coded to identify key emerging issues reported by Veterans exposed to suicide. The study is feasible and the team outlines an appropriate timeline.

Weaknesses: None identified beyond standard limitations acknowledged by the investigative team (e.g., limited ability to examine those not receiving VHA services).

4. Feasibility (including Sampling, Project Timeline and Staffing).

The proposed research strategy is well thought out and thorough, with strategic decisions well justified. The issue of power is thoroughly addressed and supports the ability to detect anticipated main effects and interaction analyses. Key issues regarding survey non-response and analytic modeling of missing data are thoroughly outlined. Proposed timeline is appropriate.

5. Implementation.

The research team has established agreements with key partners at the Office of Mental Health and Suicide Prevention (Matthew Miller, PhD., MPH), the Center for PTSD (Craig Rosen, PhD.), and the Rocky Mountain MIRECC (Suzanne McGarity, PhD. & Georgia Gerard, LICSW). These contacts will also serve as a part of the project Advisory Panel, assisting with project guidance and dissemination. The primary deliverables will include a study brief shared with key clinical stakeholders and organizations and a lay summary that can be shared with Veterans and families coping with suicide exposure.

6. Investigator Qualifications.

The research team is exceptional and well qualified to conduct the proposed research, a strong advisory panel has been established. The PI has substantial experience in leading large-scale VA research projects, mixed methods approaches, and has a stellar support team with the skillset necessary to ensure project success. The study team (and advisory panel) has experience with mixed-methods research and qualitative analyses.

7. Multiple PI Leadership Plan.

Not applicable.

8. Facilities and Resources.

The project will take place at the CCDOR of the Minneapolis VAHS and the COIN (Center for Clinical Management Research) at the Ann Arbor VAHS, and both centers have substantial research infrastructures. Appropriate documentation of support agreements has been provided.

9. Adequacy of Response to Previous Feedback Regarding the Proposed Study.

The project team has thoroughly and appropriately responded to the prior round of reviews, and the quality of the application has improved accordingly. The most important changes include: 1) The addition of two comparison groups (one exposed to sudden death other than suicide and another unexposed to any sudden loss); 2) Better specification of suicide outcome variables (e.g., ideation, planning, & attempts); and 3) The study will oversample women to examine potential sex differences. The project plan now also includes a suicide risk assessment safety plan, troubleshooting for increased survey response rates, and more attention to non-VHA enrollees in examining the data on suicide and PTSD outcomes. Overall, the application is much stronger following revision.

10. Protection of Human Subjects from Research Risk.

No concerns.

11. Inclusion of Women and Minorities in Research.

No concerns.

12. Budget and Period of Support.

No concerns.

13. Sharing Research Data (Data Management and Access Plan (DMAP).

Appropriate.

14. Overall Impression.

The proposed research examines the important and understudied topic of suicide-exposure among Veterans, and will answer key questions about how exposed Veterans respond with grief and trauma reactions and potential suicide risk concerns of their own. The focus of the study is consistent with HSR& priority areas in suicide prevention. The study will utilize three appropriately powered waves of data collection to (Wave 1) identify suicide exposure incidence and identify the key suicide-exposure group and two relevant control conditions utilized in Wave 2, and the groups will be compared on various measures of clinical function. The study also contains qualitative interviews (Wave 3) meant to help inform the major challenges Veterans exposed to suicide face. Women and Native American Veterans will be oversampled for additional information on these essential groups. Overall, the study has strong potential to increase understanding of down-stream effects of suicide exposure on the mental health of Veterans and will likely result in improved clinical care efforts. Minimal limitations or weaknesses were identified with this revision.

15. Key Strengths.

1. Sampling strategies and recruitment plans well thought out and feasible.

2. Mixed methods design including qualitative and quantitative data approaches.

3. Experience research team with necessary research skills and dissemination contacts.

4. Women and Native American Veterans will be oversampled.

5. Study has potential to identify modifiable factors that can be targeted to reduce negative outcomes.

16. Key Weaknesses.

1. Limited inclusion of Veterans not utilizing VHA services.

Footnotes for 1 I01 HX003438-01A1; PI Name: SAYER, NINA A.

+ Derived from the range of percentile values calculated for the study section that reviewed this application.

MEETING ROSTER

HSR-4 Mental and Behavioral Health
Health Services Research Parent IRG
Office of Research & Development
HSR4
08/26/2021 - 08/27/2021

CHAIRPERSON(S)

MARTIN, JENNIFER L, PHD
RESEARCH SCIENTIST
VA GREATER LOS ANGELES HEALTHCARE SYSTEM
PROFESSOR
DAVID GEFFEN SCHOOL OF MEDICINE
UNIVERSITY OF CALIFORNIA, LOS ANGELES
LOS ANGELES, CA 91343

MEMBERS

BEEHLER, GREGORY P, PHD
ASSOCIATE DIRECTOR OF RESEARCH
VA WESTERN NEW YORK HEALTHCARE SYSTEM
RESEARCH ASSOCIATE PROFESSOR
COMMUNITY HEALTH AND HEALTH BEHAVIOR
UNIVERSITY AT BUFFALO
BUFFALO, NY 14215

BLACK, ANNE C., PHD
RESEARCH HEALTH SCIENTIST
VA CONNECTICUT HEALTHCARE SYSTEM
ASSISTANT PROFESSOR
YALE SCHOOL OF MEDICINE
WEST HAVEN, CT 06516

BROWN, GREGORY K, PHD
RESEARCH CLINICAL PSYCHOLOGIST
PHILADELPHIA VAMC
RESEARCH ASSOCIATE PROFESSOR
PERELMAN SCHOOL OF MEDICINE
UNIVERSITY OF PENNSYLVANIA
PHILADELPHIA, PA 19104

BRYAN, CRAIG J., PSYD
HEALTH SCIENCE SPECIALIST
CANANDAIGUA VAMC
CENTER OF EXCELLENCE FOR SUICIDE PREVENTION,
STRESS, TRAUMA, AND RESILIENCE PROFESSOR
OHIO STATE UNIVERSITY
COLUMBUS, OH 43210

COMTOIS, KATHERINE ANNE JR, PHD
PROFESSOR
DEPARTMENT OF PSYCHIATRY & BEHAVIORAL SCIENCES
DEPARTMENT OF PSYCHOLOGY
UNIVERSITY OF WASHINGTON
SEATTLE, WA 98195

DAVIS, LORI L., MD
ASSOCIATE CHIEF OF STAFF FOR RESEARCH
TUSCALOOSA VAMC
CLINICAL PROFESSOR
DEPARTMENT OF PSYCHIATRY & BEHAVIORAL
NEUROBIOLOGY
UNIVERSITY OF ALABAMA AT BIRMINGHAM
TUSCALOOSA, AL 35404

FLETCHER, TERRI LYNN, PHD
CLINICAL RESEARCH PSYCHOLOGIST
HOUSTON VAMC
ASSOCIATE PROFESSOR
BAYLOR COLLEGE OF MEDICINE
HOUSTON, TX 77030

FRENCH, DUSTIN D., PHD
RESEARCH SCIENTIST
HINES VAMC
PROFESSOR
NORTHWESTERN UNIVERSITY
EDWARD HINES, JR VA HOSPITAL
HINES, IL 60141

HAMNER, MARK B., MD
STAFF PSYCHIATRIST
CHARLESTON VAMC
PROFESSOR
MEDICAL UNIVERSITY OF SOUTH CAROLINA
CHARLESTON, SC 29451

KIMBREL, NATHAN A., PHD
CO-DIRECTOR
DURHAM VA HEALTH CARE SYSTEM
MID-ATLANTIC MIRECC CLINICAL CORE
ASSOCIATE PROFESSOR
DUKE UNIVERSITY SCHOOL OF MEDICINE
DURHAM, NC 27705

MCINTOSH, SCOTT, PHD
RESEARCH DIRECTOR, DIVISION OF SOCIAL & BEHAVIORAL
SCIENCES
ASSOCIATE PROFESSOR
DEPARTMENT OF PUBLIC HEALTH SCIENCES
UNIVERSITY OF ROCHESTER MEDICAL CENTER
ROCHESTER, NY 14642

PEIRCE, JESSICA M, PHD
ASSOCIATE PROFESSOR
DEPARTMENT OF PSYCHIATRY AND BEHAVIORAL SCIENCES
JOHNS HOPKINS UNIVERSITY SCHOOL OF MEDICINE
BALTIMORE, MD 21224

PENFOLD, ROBERT B., PHD
SENIOR SCIENTIFIC INVESTIGATOR
KAISER PERMANENTE
WASHINGTON HEALTH RESEARCH INSTITUTE
AFFILIATE ASSOCIATE PROFESSOR, HEALTH SERVICES
UNIVERSITY OF WASHINGTON, SCHOOL OF PUBLIC HEALTH
SEATTLE, WA 98101

PRIMACK, JENNIFER MARIE, PHD
HEALTH SCIENTIST
PROVIDENCE VAMC
ASSISTANT PROFESSOR
BROWN UNIVERSITY
PROVIDENCE, RI 01772

ROLLINS, ANGELA LEIGH, PHD
SUPERVISORY RESEARCH HEALTH SCIENTIST
VETERANS HEALTH INDIANA VAMC
ASSOCIATE RESEARCH PROFESSOR
INDIANA UNIVERSITY- PERDUE UNIVERSITY INDIANAPOLIS
INDIANAPOLIS, IN 46202

SCHUMM, JEREMIAH, PHD
PROFESSOR
SCHOOL OF PROFESSIONAL PSYCHOLOGY
WRIGHT STATE UNIVERSITY
DAYTON, OH 45435-0001

SELBY, EDWARD ANDREW, PHD
ASSOCIATE PROFESSOR
DEPARTMENT OF PSYCHOLOGY
RUTGERS, THE STATE UNIVERSITY OF NEW JERSEY
PISCATAWAY, NJ 08854

SCIENTIFIC REVIEW OFFICER

O'BRIEN, ROBERT W., PHD
DEPARTMENT OF VETERANS AFFAIRS
VETERANS HEALTH ADMINISTRATION
OFFICE OF RESEARCH AND DEVELOPMENT
HEALTH SERVICES RESEARCH & DEVELOPMENT
WASHINGTON , DC 20420

Consultants are required to absent themselves from the room during the review of any application if their presence would constitute or appear to constitute a conflict of interest.
